# “I wasn’t prepared for this”: a grounded theory study of student teachers’ stressors and coping mechanisms during teaching internship in physical education

**DOI:** 10.3389/fpsyg.2026.1841886

**Published:** 2026-06-24

**Authors:** Chao Wang, Dejun Yang, Zhongming Shi

**Affiliations:** 1 Faculty of Education, Shinawatra University, Pathum Thani, Thailand; 2 College of Sports and Great Health, Sichuan Technology and Business University, Meishan, China; 3 ISEI BSU, Belarusian State University, Minsk, Belarus

**Keywords:** coping strategy, physical education, professional identity, student teachers, teaching internship stress

## Abstract

Grounded in the transactional model of stress and coping and the conservation of resources theory, this study employed a grounded theory approach to systematically investigate the stressors, coping mechanisms, and professional identity formation among elementary school physical education student teachers during their teaching internship. Semi-structured in-depth interviews were conducted with 30 student teachers in a city in western China. Using NVivo 15.0 for three-level coding analysis, an integrated theoretical model centered on “stress events, cognitive appraisal, coping strategies, adaptation outcomes, and a feedback loop” was constructed. The findings reveal that the stressors experienced by physical education student teachers are teaching centered, characterized by task complexity and interpersonal dynamics, fundamentally stemming from an imbalance between resource supply and demand. Cognitive appraisals fell into three types: challenging controllable, threatening uncontrollable, and helplessness. These appraisals, respectively, led to three categories of coping strategies: resource gain oriented, resource protection oriented, and resource depletion oriented. Positive adaptation outcomes, such as enhanced teaching competence and strengthened professional identity, or negative outcomes, including burnout and low self-efficacy, resulted from different coping strategies. Adaptation outcomes subsequently regulated subsequent cognitive and coping behaviors through a feedback mechanism. Based on these findings, the proposed interventions target four dimensions: optimizing resource supply, strengthening cognitive guidance, cultivating coping strategies, and constructing support systems. This study offers theoretical and practical support for improving the quality of physical education internships and cultivating high quality physical education teachers.

## 1 Introduction

Internship is an essential pathway for education majors to apply theoretical knowledge to teaching practice, representing a critical stage in teacher education (Cheng et al., 2012; Gill and Dalgarno, 2017). During this period, student teachers must integrate theoretical knowledge with practical skills, navigate complex school environments, and develop their professional identity (Gholami et al., 2021; Hanna et al., 2022). However, student teachers across various disciplines experience significant stress during their internships, stemming from a complex combination of classroom management difficulties, workload pressures, and the nature of mentoring relationships (Gholami et al., 2021). In the specific context of physical education, these stressors are amplified by the unique characteristics of the discipline: outdoor teaching environments accompanied by variable weather conditions, heightened safety concerns regarding student injuries, physical exertion during skill demonstration, and the marginalized status of physical education within the school curriculum (Mckown et al., 2025; Shume and Blatt, 2019). The accumulation of these stressors not only threatens the mental health of student teachers but may also undermine their commitment to the teaching profession, exacerbating the globally recognized issue of teacher attrition (Juul et al., 2021; Körkkö et al., 2025). Therefore, understanding the nature of the stressors encountered by physical education student teachers and how they cope with these challenges has become an urgent issue for both teacher educators and policymakers (Sakalli and Senel, 2025).

Although a large number of scholars have conducted research on the sources of stress and coping strategies among student teachers in physical education, several limitations remain in the existing literature. First, most studies employ quantitative methods using predetermined stress and coping scales, which may fail to capture the dynamic and context-dependent nature of the coping process as it unfolds over time (Chen et al., 2025). Second, the application of these theories to the specific population of student teachers remains insufficient; in particular, how the unique developmental stage of internship shapes stress appraisal and resource mobilization has not been thoroughly explored (Homann et al., 2024). Third, and most critically, the vast majority of studies utilizing these frameworks have been conducted in Western educational contexts, and their applicability across different cultural backgrounds, educational systems, and policy environments worldwide remains inadequately examined. From a global perspective, non-Western countries and regions differ significantly from Western contexts in terms of educational philosophies, teacher-student relationships, and school culture, yet research on physical education student teachers in these settings is extremely scarce (Ellison and Walton-Fisette, 2022; Grstn and Kokkonen, 2021). These research gaps highlight the necessity of conducting qualitative, context-sensitive studies across the globe to enrich the understanding of stress and coping among physical education student teachers in diverse cultural contexts.

Given the critical transition represented by the teaching internship, the unique stressors inherent to physical education, and the global teacher attrition crisis, understanding stress and coping among PE student teachers is both theoretically and practically imperative (Kim M. et al., 2025). However, existing research suffers from methodological over-reliance on static quantitative designs, insufficient integration of the transactional model of stress and coping with conservation of resources theory for this specific population, and a striking neglect of non-Western educational contexts—particularly elementary school PE internships in underdeveloped regions such as Western China (Fan and Xie, 2025). Focusing on elementary school PE student teachers is especially warranted because this stage is pivotal for children’s physical literacy development, presents heightened classroom management challenges due to young learners’ developmental characteristics, and occurs in resource-scarce settings where interns often lack systematic support. To address these gaps, this study offers four unique contributions. First, it systematically integrates Lazarus and Folkman’s transactional model with Hobfoll’s conservation of resources theory into a unified theoretical framework (Lazarus and Folkman, 1984; Hobfoll, 1989). Second, it employs a grounded theory approach to capture dynamic, context-dependent processes of stress and coping. Third, it investigates an under-researched population in Western China, thereby providing much-needed contextual evidence. Fourth, it constructs a feedback-loop model that reveals stress adaptation as an iterative, intervenable cycle. The expected benefits are twofold: theoretically, it extends both theories to the PE internship context and proposes a novel tripartite coping classification; practically, it informs pre-internship curriculum design, support system construction in internship schools, and policy development for improving internship quality in under-resourced regions.

To systematically investigate the sources of stress and coping strategies among physical education student teachers, this study draws on two complementary theoretical perspectives: the transactional model of stress and coping (Lazarus and Folkman, 1984), and the conservation of resources theory (Hobfoll, 1989). The transactional model posits that stress does not arise solely from external events but is generated through the interaction between the individual and the environment. In this process, we understand stress through a two-step cognitive appraisal: first, making a preliminary judgment about whether a given situation poses a threat or a challenge; then, evaluating what resources and methods are available to cope with it (Lazarus and Folkman, 1984). The conservation of resources theory complements this cognitive orientation from a motivational perspective, suggesting that stress occurs when individuals’ valued resources—such as self-efficacy, social support, and institutional support—are threatened or fail to yield expected returns after investment (Hobfoll, 1989). These two theories have been widely applied in physical education teacher education research. Studies grounded in the transactional model have revealed how physical education teachers appraise challenges such as classroom management difficulties, insufficient student engagement, and professional marginalization (Pérez-Salas et al., 2025; Schlesier and Westphal, 2024; Trigueros et al., 2025), while research adopting the resource-based perspective has identified the protective roles of mentor support, collegial relationships, and school climate in mitigating teacher stress and burnout (Lang et al., 2019; Melguizo-Ibáñez et al., 2023). This study employs a grounded theory approach to explore the stressors and coping mechanisms of physical education student teachers in a city in Western China during their teaching internship, capturing the complexity and processual nature of stress and coping from the participants’ perspectives while remaining open to the emergence of new concepts that may extend existing theories. Through in-depth, semi-structured interviews with physical education student teachers, this study aims to address the following research questions: *RQ1*. What stressors do physical education student teachers encounter during their teaching internship? *RQ2*. How do they cope with these stressors? *RQ3*. How do these experiences of stress and coping influence their professional identity formation? Through a systematic examination of these questions, this study seeks to propose a substantive strategic framework for coping with stressors applicable to physical education student teachers globally.

## 2 Research design

### 2.1 Research method

**Figure 1:**
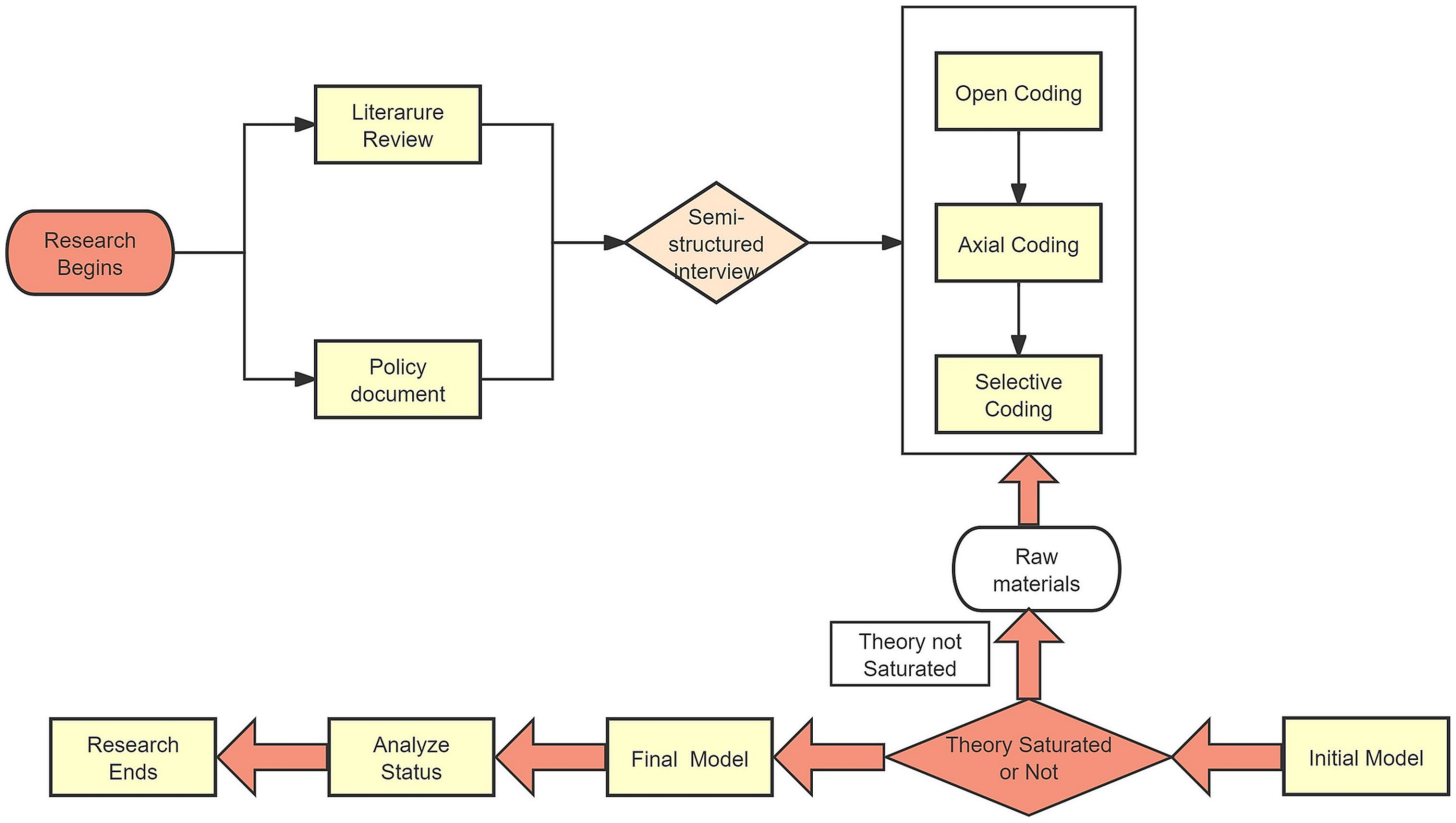
Research process.

This study employs a grounded theory approach to conduct qualitative research, drawing on the transactional model of stress and coping and the conservation of resources theory to explore the stressors and coping strategies faced by physical education student teachers in a city in Western China, as well as how these experiences of stress influence their professional identity. Semi-structured interviews were used to collect data, as this method ensures that the research focus remains aligned with the predefined interview guide while providing interviewers with sufficient flexibility to pose in-depth follow-up questions based on participants’ responses, thereby uncovering authentic and rich information beyond the preset framework (Wei et al., 2024). After collecting the interview data, the research team guided the analysis process using the three-level coding of grounded theory—namely open coding, axial coding, and selective coding—and employed triangulation throughout the analysis to enhance the reliability and validity of the study and to test theoretical saturation. The specific process is illustrated in Figure 1.

### 2.2 Research subjects and sampling method

In the initial stage of participant recruitment, this study adopted maximum variation sampling to capture the diversity of elementary school physical education student teachers across key characteristics. The sampling criteria included participants’ gender (male/female), educational background (associate degree/bachelor’s degree), motor skill level (excellent/good/average), location of the internship school (urban/suburban/rural), and the quality of their relationship with the mentor teacher (close/moderate/distant). The research team first selected four public elementary schools (coded A, B, C, and D) in a city in Western China. Through personal networks and recommendations from internship supervisors, student teachers with diverse backgrounds were deliberately recruited from each school according to the above criteria. A total of 12 participants were initially recruited through maximum variation sampling (Patton, 1990), three from each school, with equal gender distribution and variation in skill level and mentor relationship quality.

After completing interviews and preliminary analysis with the initial group of participants, the research team employed snowball sampling by asking the interviewed student teachers to recommend other eligible peers who were willing to share their authentic experiences (Coleman, 1958; Goodman, 1961). An additional 18 physical education student teachers were recruited through this method. The recruitment process continued until theoretical saturation was achieved. Ultimately, a total of 30 elementary school physical education student teachers (15 female, 15 male) were included in this study.

### 2.3 Interview outline and structure

This study developed the interview outline by reviewing existing literature and relevant policy documents. The outline is divided into seven sections, aligned with the research themes and the transactional model of stress and coping—conservation of resources theory. In brief, Section A, titled “Opening Guidance,” aims to capture participants’ primary appraisals of their internship experience—how they perceive the overall experience as positive, negative, or complex—and employs the critical incident technique, asking participants to recall a memorable event from their internship (Zhao et al., 2021). Section B, titled “Internship Expectations and Reality Shock,” seeks to understand how participants compare their actual experiences with their initial expectations (Ha and Dakich, 2022). According to the transactional model of stress and coping, stress arises from an individual’s appraisal of environmental demands (Lazarus and Folkman, 1984). When a significant discrepancy emerges between expectation and reality, the individual must reappraise the situation, a process often accompanied by intense emotional experiences. Section C, titled “Key Stressful Events and Challenges,” aims to identify the specific stressful events encountered by student teachers (Schlesier and Westphal, 2024). Section D, titled “Coping Strategies and Resource Utilization,” seeks to understand the specific actions participants take when facing stress (Gagnon et al., 2019). According to the transactional model, coping strategies can be categorized as problem-focused coping (directly addressing the stressor) and emotion-focused coping (managing emotional responses). In line with conservation of resources theory, individuals mobilize various resources to cope under stress. Section E, titled “Key Figures and Interpersonal Interactions,” examines the role of interpersonal relationships of physical education student teachers in the stress and coping process (Daugherty, 2011). Section F, titled “Growth, Reflection, and Professional Identity,” aims to understand how experiences of stress and coping shape student teachers’ professional identity and career intentions (van Heijst et al., 2024; Wu et al., 2024). According to conservation of resources theory, prolonged resource depletion may lead to burnout and turnover intentions, whereas successful resource accumulation enhances professional commitment (Hobfoll, 1989). Section G, titled “Conclusion,” provides participants with a final opportunity to add any content they consider important but that has not been addressed (Osterlie, 2025). This also reflects the participant-led principle in grounded theory research.

### 2.4 Interview procedure

Each interview was designed to last 45–75 min, with the process emphasizing the protection of participants’ psychological safety and ensuring that they retained the initiative in disclosing information. Before each interview, the interviewer reiterated the research purpose, confidentiality measures, and the principle of voluntary participation, including the participants’ right to skip questions or withdraw from the interview at any time. After obtaining the participants’ consent, the interview was audio-recorded to facilitate subsequent transcription and analysis. The interviewer employed neutral, non-leading probing techniques to clarify participants’ sources of stress, their coping strategies, and their professional identity. Following each interview, the interviewer compiled field notes, documenting non-verbal information (such as moments of hesitation or emotional fluctuations) and analytical reflections to support subsequent coding.

### 2.5 Data management

After each semi-structured interview, the audio recordings were transcribed verbatim into textual data, which then underwent rigorous anonymization (replacing names with participant codes P1–P30) and de-identification to protect participant privacy. The resulting transcripts were imported into the qualitative data analysis software NVivo 15.0 for systematic data management and analysis. The entire process from raw data to emerging themes was documented through coding manuals and memos to enhance the trustworthiness of the study.

### 2.6 Data analysis and coding

This study employed grounded theory to guide the data analysis process. The data analysis followed a three-level coding sequence: open coding, axial coding, and selective coding (Glaser and Strauss, 1967).

The first step was open coding, which involved examining the aforementioned textual data word by word and sentence by sentence, while selecting relevant content for documentation and conducting preliminary conceptual summarization. The second step was axial coding, aimed at categorizing and integrating the concepts formed during the open coding phase, establishing logical relationships among them, and forming categories at a more abstract level. The core tasks at this stage were to identify causal relationships, contextual conditions, interaction strategies, and outcome consequences among the concepts. The final step was selective coding, which aimed to identify a core category among all categories and construct a complete theoretical framework around it. The core category should be able to encompass all other categories and explain the main narrative of the research phenomenon.

To ensure the reliability and validity of the study, a rigorous dual-coding and cross-validation mechanism was implemented throughout the coding process. First, two researchers on the team with experience in qualitative research independently coded 20 randomly selected interview transcripts simultaneously. Second, an expert with grounded theory expertise was invited to code the remaining 10 interview transcripts. Finally, the three researchers compared and discussed their respective results until consensus was reached (Strauss and Corbin, 1990; Lincoln and Guba, 1985). Additionally, the research team employed an iterative validation model of “data—coding—theory,” continuously tracing back to the original data. When no new core categories emerged, theoretical saturation was considered achieved.

## 3 Grounded theory coding results

This coding process strictly adhered to the transactional model of stress and coping and the conservation of resources theory, deconstructing the internship process into four core modules: “stress events—cognitive appraisal—coping strategies—adaptation outcomes.” The results of open coding, axial coding, and selective coding are presented sequentially below.

### 3.1 Open coding

Open coding is the process of carefully analyzing phenomena or data, breaking down raw materials into basic concepts, and then naming and categorizing these concepts. The research team transcribed the 20 audio recordings into text and imported the transcripts into NVivo 15.0. With an open stance, they conducted a word-by-word and sentence-by-sentence analysis of the interview transcript raw data, conceptually refining the raw data, integrating concepts sharing the same essential attributes into conceptual sets within the same category, and extracting initial categories. Through open coding, 42 initial concepts were ultimately formed, and 7 basic categories were refined. To clearly express the coding results, this section will present the results in textual description.

Stress sources: Teaching Practice Conflict, Classroom Disorder, Individual Differences Among Students, Work Overload, Competition/Activity Pressure, Interpersonal Interaction Friction, Professional Cognition Gap, Sudden Situation Impact.

Cognitive appraisal: Threat Appraisal, Challenge Appraisal, Powerlessness Appraisal, Controllable Appraisal, Uncontrollable Appraisal, Value Relevance Appraisal.

Problem-focused coping: Skill Improvement Actions, Teaching Adjustment and Optimization, Task Management and Planning, Interpersonal Communication Improvement, Proactive Resource Acquisition.

Negative coping: Giving-Up Tendency, Self-Denial, Emotional Suppression, Interpersonal Alienation.

Positive adaptation outcomes: Teaching Ability Improvement, Professional Cognition Deepening, Mature Mental Model, Professional Identity Enhancement.

Negative adaptation outcomes: Harmonious Interpersonal Relationships, Job Burnout Tendency, Low Self-Efficacy, Interpersonal Alienation and Avoidance.

### 3.2 Axial coding

Axial coding utilized the node association function of NVivo 15.0 to perform logical clustering of the basic categories formed during open coding, ultimately resulting in four main categories and constructing a theoretical framework of stressors and coping strategies for physical education student teachers. The coding logic and conceptual interpretation of each main category are presented in Table 1.

**Table 1:** Axial coding results.

Main category	Core connotation	Logical position
Internship stress events	“Environmental input” for transactional interaction	Stressors (environmental factors)
Cognitive appraisal process	The core intermediary connecting “stress events” and “coping strategies”	Cognitive interaction (primary + secondary appraisal)
Transactional coping strategies	The core behavioral performance of “individual-environment” transactions, divided into two categories: “changing the event” and “regulating emotions”	Coping behaviors (core of transactions)
Adaptive outcome output	The adaptive state achieved between the individual and the environment is the ultimate product of transactional interaction	Adaptive outcomes (products of transactions)

### 3.3 Selective coding

In this study, selective coding was guided by the transactional model of stress and coping and the conservation of resources theory to distill the core category—“The Transaction and Adaptation Process of Stress in Physical Education Internship”—and to construct a complete transactional storyline around this core category, clarifying the logical relationships between the core category and the main categories, as shown in Table 2.

**Table 2:** Selective coding results.

Main category	Transactional logical relationship with the core category	Model mechanism explanation
Internship stress events	The “starting point of environmental transactions”	The “input variable” in the transaction process
Cognitive appraisal process	The “core of transactional intermediary”	Reflects the subjective interaction between “individual and environment”
Transactional coping strategies	The “carrier of transactional behaviors”	The “core action variable” in the transaction process
Adaptive outcome output	The “product of transactional interactions” of the core category, and the “feedback variable” for the next round of transactions	It is the ultimate result of transactional interaction

The core category in this study is defined as “The Transaction and Adaptation Process of Stress in Physical Education Internship.” This refers to the process through which physical education student teachers, situated within the internship environment, continuously interact with various stressful events (Kim Y. et al., 2025). They interpret the nature and controllability of stress through cognitive appraisal, subsequently select problem-focused or emotion-focused coping strategies, and ultimately achieve either positive or negative adaptation outcomes (Gloria and Steinhardt, 2016). Simultaneously, these adaptation outcomes influence subsequent cognitive appraisals and coping strategy selections through a feedback mechanism, forming a dynamic transactional cycle of “stress events—cognitive appraisal—coping strategies—adaptation outcomes—reappraisal.”

Upon entering the internship environment, physical education student teachers first encounter objective stressful events such as conflicts in teaching practice and loss of classroom order (Yoon et al., 2024). Through primary appraisal, they judge the relevance of these events to their professional growth; through secondary appraisal, they assess the nature of the events and their controllability (Fletcher and Beckey, 2023). Based on this cognitive appraisal, they select corresponding transactional coping strategies. If the cognitive appraisal process yields a “challenging + controllable” evaluation, they tend to adopt problem-focused coping strategies such as skill enhancement and teaching optimization. If the appraisal yields a “threatening + uncontrollable” evaluation, they tend to adopt emotion-focused coping strategies such as emotional release and seeking social support. Following the implementation of coping strategies, different adaptation outcomes emerge: positive coping generally leads to positive outcomes such as enhanced teaching ability and strengthened professional identity (Lee and Janke, 2025; Wong and Liu, 2024), whereas negative coping tends to result in negative outcomes such as burnout and low self-efficacy (Zheng et al., 2023). Ultimately, these adaptation outcomes influence subsequent cognitive appraisals through a feedback mechanism, thereby shaping the selection of coping strategies in response to the next round of stressful events, forming a dynamic cyclical process of stress transaction and adaptation (van der Linden et al., 2018).

## 4 Theoretical model construction

**Figure 2:**
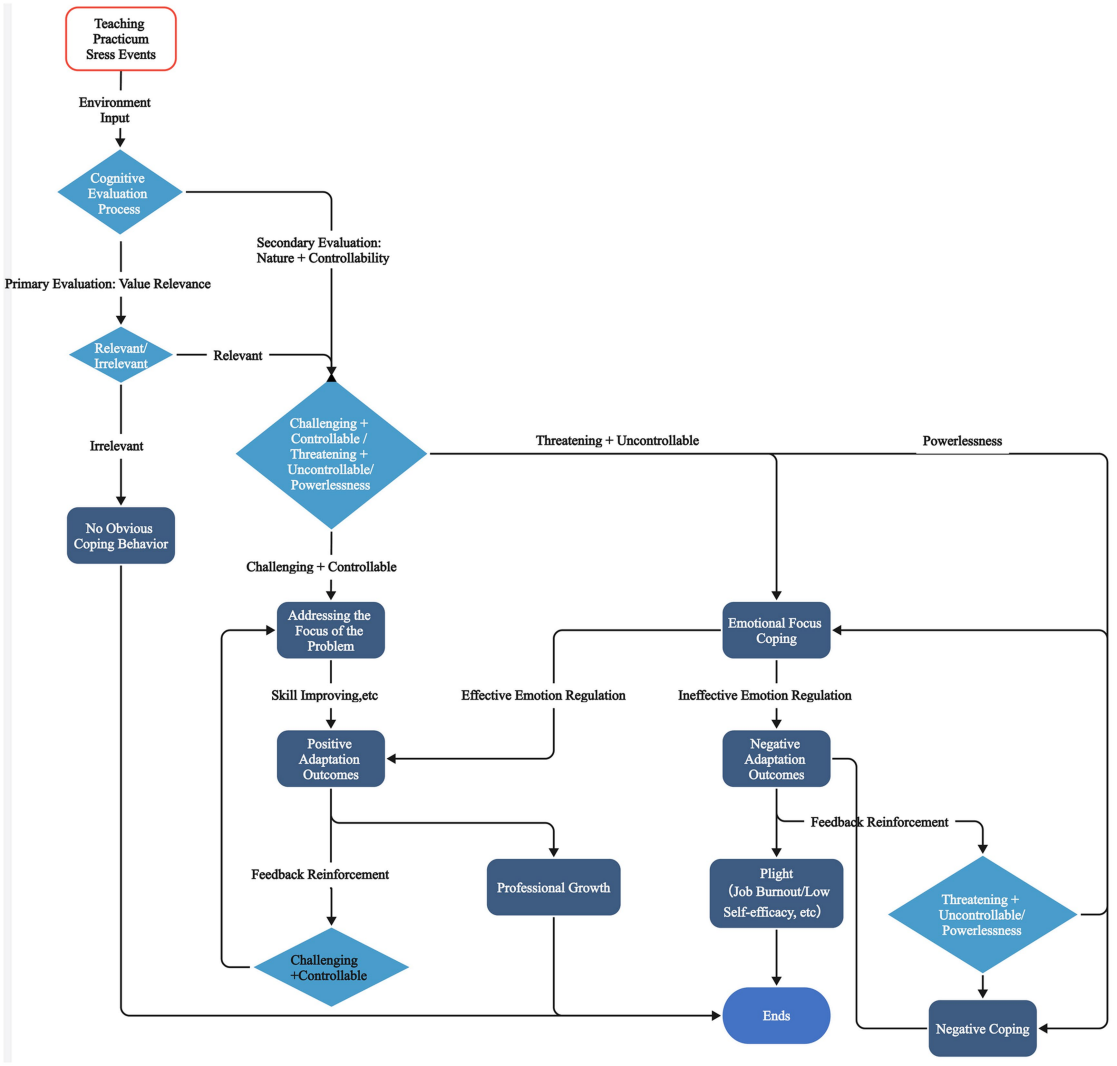
Theoretical model.

The core logic of the transactional model of stress and coping is that stress is a dynamic process of interaction between the individual and the environment. The individual interprets the stressful events encountered and their controllability through cognitive appraisal, thereby selecting strategies to cope with the stress, ultimately leading to adaptation outcomes (Lazarus and Folkman, 1984). The central tenet of the conservation of resources theory is that stress arises from resource loss and the failure to achieve resource gains, while effective coping behaviors are essentially the acquisition, integration, and utilization of resources (Hobfoll, 1989). Through grounded theory coding of interview data from 20 physical education student teachers, this study reveals the dynamic mechanisms and adaptation pathways of internship stress. The findings show that stress among physical education student teachers originates from objective events such as conflicts in teaching practice, loss of classroom order, and excessive workload. Through primary and secondary appraisal, student teachers form cognitive interpretations characterized as threatening, challenging, or powerlessness, and subsequently select problem-focused coping, emotion-focused coping, or negative coping. Different coping strategies ultimately lead to positive outcomes such as enhanced teaching ability and strengthened professional identity, or negative outcomes such as burnout and low self-efficacy. Adaptation outcomes then, through a feedback mechanism, inversely influence subsequent cognitive appraisals and coping strategy selections, forming a dynamic cycle of “stress events—cognitive appraisal—adaptation outcomes—reappraisal.” Based on the aforementioned theoretical foundations and empirical findings, an integrated theoretical model of stressors and coping adaptation strategies for physical education student teachers was constructed, as shown in Figure 2.

As illustrated in the integrated theoretical model, coping strategies serve as the core behavioral mechanism in the stress transaction process and directly determine the direction and effectiveness of adaptation. In line with the transactional model of stress and coping (Lazarus and Folkman, 1984), three distinct coping strategies were identified and conceptualized in this study, reflecting different approaches to stressors and resource management.

### 4.1 Problem-focused coping

Problem-focused coping refers to adaptive, approach-oriented behavioral and cognitive efforts aimed at managing, altering, or directly resolving the source of stress (Pogere et al., 2019). It targets the root cause of the stressful situation by reducing external demands, mobilizing resources, improving professional skills, or optimizing teaching practices, thereby weakening or eliminating the impact of the stressor itself.

### 4.2 Emotion-focused coping

Emotion-Focused coping involves adaptive cognitive and behavioral responses designed to regulate negative emotions, psychological distress, and affective arousal caused by stressors, rather than changing the stressful event itself (Leslie-Miller et al., 2025). It functions to alleviate emotional discomfort, restore emotional equilibrium, protect psychological resources, and maintain individual functioning.

### 4.3 Negative coping

Negative coping constitutes maladaptive, avoidance-oriented responses characterized by behavioral disengagement, emotional suppression, self-denial, withdrawal, and inaction. Such strategies fail to address stressors or regulate emotions effectively, deepen psychological strain, accelerate resource depletion, and hinder normal adaptive processes (Nowacki and Pyszkowska, 2024).

## 5 Discussion

This section discusses the three core research questions proposed in this study, namely the primary stressors encountered by physical education student teachers during their teaching internship (RQ1), their coping strategies in response to these stressors (RQ2), and the influence of stress and coping experiences on the formation of their professional identity (RQ3). Through sequential analysis and integration of the above questions, this section aims to reveal the underlying mechanisms of the internship stress coping process, engage in dialogue with existing theoretical and empirical research, and provide practical implications for improving the quality of physical education internships.

*RQ1*: The study finds that the stress experienced by physical education student teachers during their internship is characterized by multi-dimensional features: teaching-centeredness, task complexity, and interpersonal dynamics. Conflicts in teaching practice, loss of classroom order, and individual student differences constitute the core stressors, which are closely related to the unique characteristics of physical education, such as outdoor teaching, physical engagement, and difficulty in discipline control (Emslander et al., 2025; Li et al., 2022; Watanabe, 2013). Excessive workload and interpersonal friction form significant stressors. Discrepancies in professional expectations and unexpected incidents serve as additional sources of stress. From the perspective of conservation of resources theory, these stressors represent an imbalance between “resource possession” and “resource demand.” The professional resources required during the internship, such as instructional design, classroom management, and interpersonal communication, along with psychological resources like time management and emotional regulation, are in sharp conflict with the student teachers’ resource deficits. The intensity of stress is directly determined by the degree of this imbalance between resource supply and demand. This finding validates the core tenet of conservation of resources theory that “stress arises from resource loss or the failure to achieve resource gains.”

*RQ2*: Cognitive appraisal, as the core mediator in the transactional model of stress and coping, demonstrates a clear categorization and guiding function in this study. Through primary and secondary appraisal, student teachers interpret the stress they experience into three appraisal outcomes: challenging + controllable, threatening + uncontrollable, and powerlessness. These different appraisal outcomes directly determine the selection of distinct coping strategies (Dekeyser et al., 2025). Corresponding to the three types of cognitive appraisal outcomes, student teachers adopt three categories of coping strategies: resource-gain-oriented, resource-protection-oriented, and resource-depletion-oriented. Problem-focused coping, which actively acquires resources to address the root cause of stress through methods such as enhancing teaching abilities and optimizing instruction, falls under resource-gain-oriented coping. Emotion-focused coping, which protects existing resources from depletion through methods such as emotional release and seeking social support, falls under resource-protection-oriented coping. Negative coping, such as self-denial and interpersonal withdrawal, leads to excessive resource depletion and falls under resource-depletion-oriented coping. This finding not only aligns with the dynamic logic in the transactional model of stress and coping that “cognitive appraisal determines coping strategies” but also explains the essence of coping strategies through conservation of resources theory, revealing the intrinsic connection between cognition, coping, and resources.

*RQ3*: The study confirms that different coping strategies directly lead to divergent adaptation outcomes. Student teachers who predominantly adopt problem-focused coping strategies, supplemented by emotion-focused coping strategies, can achieve comprehensive gains in professional resources, cognitive resources, psychological resources, and social resources, ultimately resulting in positive adaptation outcomes characterized by strengthened professional identity. Conversely, negative coping strategies lead to continuous resource loss, triggering negative adaptation outcomes such as burnout, low self-efficacy, and interpersonal withdrawal and avoidance. More importantly, adaptation outcomes inversely influence subsequent cognitive appraisals and resource states through a feedback mechanism. Positive adaptation outcomes reinforce challenging + controllable appraisals, forming a virtuous cycle of resource gain—positive cognition—effective coping—further resource gain. Negative adaptation outcomes reinforce threatening + uncontrollable appraisals or feelings of powerlessness, forming a vicious cycle of resource loss—negative cognition—ineffective coping—further resource loss. This closed-loop mechanism not only validates the dynamic interaction logic of the transactional model of stress and coping but also, through the resource cycle perspective of conservation of resources theory, fully presents the long-term developmental trajectory of stress coping and adaptation among physical education student teachers, highlighting the importance of early cognitive guidance and coping strategy cultivation for student teachers.

Based on the aforementioned findings, and in conjunction with the transactional model of stress and coping and the conservation of resources theory, targeted countermeasures are proposed from four dimensions: optimizing resource supply, strengthening cognitive guidance, cultivating coping strategies, and building a support system. These aim to alleviate stress among physical education student teachers globally and promote positive adaptation.

*Optimizing resource supply.* Teacher education in colleges and universities should strengthen the function of pre-service resource reserve for physical education interns (Herold and Waring, 2018). Considering the unique characteristics of the physical education discipline, it is recommended that universities offer specialized courses such as Classroom Management Practice, Teaching Competence in Motor Skill Transformation, and Event Organization and Safety Prevention. Through methods like simulated teaching and case analysis, professional resources can be provided to student teachers in advance. Additionally, courses on professional cognition and psychological adjustment should be introduced to correct idealized perceptions among physical education students and enhance psychological resources such as emotional regulation. Internship schools should reasonably control the intensity of resource demands, avoid assigning excessive administrative and extra tasks to student teachers, and protect their core time for lesson preparation and study (Gibbons et al., 2010). They should also improve teaching resource support by providing access to exemplary teaching case libraries, complete teaching equipment, and venue coordination assistance to reduce the cost of resource acquisition. A one-on-one mentorship mechanism between supervising teachers and student teachers should be established, offering regular professional guidance to rapidly supplement the practical resources of student teachers.

*Strengthening cognitive guidance.* Universities should implement cognitive interventions for student teachers both before and during the internship. By sharing the internship experiences of previous student teachers, they can help current student teachers understand the inevitability and developmental value of internship stress, guiding them to interpret stressors as challenging and controllable events (Johnson and Heidorn, 2014). Internship supervising teachers should reinforce cognitive feedback during daily guidance, helping student teachers analyze the nature of stressful events and potential solutions. Timely recognition of student teachers’ progress and emphasizing the value relevance of stressful events—through effective formative evaluation—can help cultivate positive cognitive appraisal habits.

*Cultivating coping strategies.* Student teachers should be guided to develop an integrated coping system centered on problem-focused coping, supplemented by emotion-focused coping. Universities can teach problem-focused coping techniques through specialized training and skill practice, while also imparting effective emotion-focused coping methods such as physical release, reflective writing, and seeking social support, thereby avoiding negative coping (Waterhouse and Samra, 2026). Student teachers should be trained to adopt problem-focused coping as their core strategy, devising targeted solutions for specific stressful events, and resolutely avoiding negative coping to prevent continuous resource loss.

*Building a support system.* A three-dimensional social support system encompassing the university, internship school, and family should be established. Universities should set up internship tracking mechanisms, using regular follow-ups and online communication to stay informed about student teachers’ stress levels and needs, providing targeted guidance. Internship schools should foster a positive interpersonal atmosphere, encourage collaboration and sharing among student teachers, avoid unhealthy competition, and enrich their social resources (Homann et al., 2024). Student teachers should be guided to proactively seek emotional support from their families, supplementing their psychological resources and forming a comprehensive support network (Chen and Murray, 2026). Internship schools and supervising teachers should promptly affirm student teachers’ positive performance, such as teaching improvements and enhanced interpersonal skills, reinforcing their sense of professional achievement and self-efficacy. Universities can amplify the demonstrative effect of positive adaptation outcomes through activities such as internship excellence awards and experience sharing. Student teachers can strengthen positive cognition through self-affirmation, thereby promoting the formation of a virtuous cycle.

## 6 Limitations and future research

This study has several limitations. It relies on a geographically restricted sample of 30 physical education interns from one city in Western China, limiting the generalizability of findings. The cross-sectional retrospective design depends on participants’ memory recall, which may introduce bias. Macro contextual factors such as school management systems, policy environments, and cultural values are underexplored.

Future research can address these limitations by expanding sampling across diverse regions and cultural contexts to enhance external validity. Adopting longitudinal mixed-methods will capture real-time dynamics of stress, coping, and professional identity evolution. Integrating institutional and cultural variables will provide holistic insights into the internship process. Exploring subgroup differences (e.g., gender, prior experience, sports specialization) and tracking post-internship outcomes (e.g., job satisfaction, burnout) will further strengthen theoretical depth and inform targeted support strategies for physical education student teachers.

## 7 Conclusion

The stress coping and adaptation process of physical education student teachers is a dynamic cycle of “stress events—cognitive appraisal—coping strategies—adaptation outcomes—feedback loop,” with its core lying in the balance of resource supply and demand, as well as the formation of positive cognition and effective coping. By integrating the transactional model of stress and coping with the conservation of resources theory, this study reveals the underlying mechanisms and professional characteristics of this process. The targeted countermeasures proposed—focusing on the four dimensions of resource supply, cognitive guidance, coping strategies, and support system—aim to optimize student teachers’ resource status, shape positive cognition, and cultivate effective coping abilities, ultimately promoting their professional growth and the strengthening of professional identity. This provides both theoretical and practical support for improving the quality of physical education internships and cultivating high-quality physical education teachers.

## Data Availability

The raw data supporting the conclusions of this article will be made available by the authors, without undue reservation.
