# Correction: “I wasn't prepared for this”: a grounded theory study of student teachers' stressors and coping mechanisms during teaching internship in physical education

**DOI:** 10.3389/fpsyg.2026.1930852

**Published:** 2026-07-21

**Authors:** Frontiers Production Office

**Affiliations:** Frontiers Media SA, Lausanne, Switzerland

**Keywords:** coping strategy, physical education, professional identity, student teachers, teaching internship stress

Author Chao Wang was erroneously included as corresponding author. The correct corresponding author(s) is Dejun Yang.

The original version of this article has been updated.

