# Supplementary material for: “I wasn’t prepared for this”: a grounded theory study of student teachers’ stressors and coping mechanisms during teaching internship in physical education
Source: Front Psychol. 2026 Jun 24;17:1841886. doi: 10.3389/fpsyg.2026.1841886 (PMC13341731; doi:10.3389/fpsyg.2026.1841886)
Supplement: Supplementary file 1 [file Table_1.DOCX]

**Internship Stress and Coping Strategies of Physical Education Majors**

**Semi-structured Interview Outline**

**【Opening Guidance】**

1. Thank you very much for your willingness to share your internship experience. Please recall first—if you had to use a few words or a sentence to describe your entire educational internship, what would it be? And why?

2. Please talk about your internship story from start to finish. From the moment you learned about your internship arrangement to the end of the internship, what impressive things happened in between?

**【Core Interview Questions】**

Section A: Internship Expectations and Reality Shock

3. Before you actually stepped into the school and stood on the podium, what imaginations or expectations did you have about the internship life of a physical education teacher? After you officially started the internship, what things in the first few weeks made you think, "I didn't expect it to be like this"?

4. Do you still remember your first lesson? How did you feel then? What impressive things happened during or after the lesson?

**【Section B: Key Stressful Events and Challenges】**

5. During the entire internship, was there any moment or incident that made you feel extremely difficult, stressed, or even want to escape? Please describe the situation in detail (e.g., during the lesson, lesson preparation, or interacting with teachers and students?).

6. Beyond the classroom itself, were there any other aspects of the internship that troubled you or caused stress? Such as relationships with supervising teachers and students, the school environment, or daily trivial matters?

7. Faced with these difficulties and pressures, have there been any changes in your physical state, emotions, or thoughts? (e.g., insomnia, reluctance to speak, self-doubt, etc.)

**【Section C: Coping Strategies and Resource Utilization】**

8. When you felt extremely stressed, what did you usually do to cope? You can talk about both your immediate reactions at that time and subsequent adjustments.

9. Did you take the initiative to seek help during the internship? If yes, who did you turn to (e.g., leading teachers, internship peers, family members)? What kind of support did they provide you? If no, what stopped you from asking for help?

10. Have you observed how other interns coped with difficulties? Did their methods inspire or influence you in any way?

**【Section D: Key Figures and Interpersonal Interaction】**

11. Please talk about your supervising teacher. What kind of influence did he/she have on you during the entire internship? Is there any communication or guidance that you still remember to this day (whether positive or negative)?

12. What was your relationship with the students like? Was there any interaction with students that had a profound impact on you?

13. What about your internship peers? Did you support each other, work on your own, or have a competitive relationship?

【Section F: Growth, Reflection and Professional Identity】

14. Looking back on the entire internship, what experiences have given you a new understanding of the profession of "physical education teacher"? Is this understanding positive or negative?

15. After going through these pressures and difficulties, what do you think is the biggest change between you now and you before the internship? (e.g., way of looking at problems, self-confidence, attitude towards the profession, etc.)

16. If you could give a piece of advice to the juniors and seniors who are about to take part in the internship next year, what would you say?

**【Closing】**

17. Apart from what we have talked about just now, regarding stress and coping in the internship, is there anything important that I haven't asked and you would like to add?

18. One last question: After going through all this, do you still think you will choose to be a physical education teacher in the future? Why?
