# Supplementary material for: “I wasn’t prepared for this”: a grounded theory study of student teachers’ stressors and coping mechanisms during teaching internship in physical education
Source: Front Psychol. 2026 Jun 24;17:1841886. doi: 10.3389/fpsyg.2026.1841886 (PMC13341731; doi:10.3389/fpsyg.2026.1841886)
Supplement: Supplementary file 2 [file Table_2.DOCX]

*Open Coding Results*

| Original Sentence | Initial Concept | Basic Category |
| --- | --- | --- |
| "When encountering problems that I can't solve by myself, I take the initiative to consult the supervisor teacher, who will give me specific suggestions and methods." | Teaching Practice Conflict | Stress Sources |
| "I will go to the court to play ball to vent my emotions. After sweating a lot, the pressure will be relieved a lot." | Classroom Disorder |  |
| "Give myself positive hints, telling myself that it's just lack of experience, and don't deny everything because of one poorly taught class." | Individual Differences Among Students |  |
| "Listen to soothing music or dance to music for a while to relax myself." | Work Overload |  |
| "Write a simple summary to sort out the chaotic ideas. Even a few lines can help me sort out my thoughts quickly." | Competition/Activity Pressure |  |
| "Call my parents to listen to their encouragement. Family support is a very important spiritual pillar for me." | Interpersonal Interaction Friction |  |
| "After that class, I was exhausted and felt extremely frustrated. I thought I couldn't even organize a simple small game, so maybe I wasn't qualified to be a physical education teacher at all. I didn't have the energy to teach for several days." | Professional Cognition Gap |  |
| "The utilitarianism and internal friction of this profession make me see no hope. I want to find a more humanistic job and no longer want to insist on being a physical education teacher." | Sudden Situation Impact |  |
| "When under great pressure, I become particularly emotionally sensitive and cry over small things, but I don't want to tell anyone and just keep it to myself." | Threat Appraisal | Cognitive Appraisal |
| "I was always afraid of saying the wrong thing when communicating with the supervisor teacher, so I tried to speak as little as possible, rarely consulted, and took on problems by myself. Gradually, the relationship with the teacher became very distant." | Challenge Appraisal |  |
| "I have become more calm and patient, no longer as impetuous as before. My classroom organization and communication skills have also improved a lot." | Powerlessness Appraisal |  |
| "I found that physical education teachers are not only transmitters of sports skills, but also guides for students' growth, who need to balance teaching and educating people." | Controllability Appraisal |  |
| "My mentality has become more mature. From avoiding problems to solving them calmly, my self-confidence has increased, and my professional attitude has changed from 'giving it a try' to 'wanting to do it well'." | Uncontrollability Appraisal |  |
| "Seeing the students' progress and feeling needed, I have established a solid professional self-confidence and believe that I can become a qualified physical education teacher." | Value Relevance Appraisal |  |
| "Established good relationships with supervisor teachers and internship peers, and can support and help each other when encountering problems." | Skill Improvement Actions | Problem-Focused Coping |
| "The utilitarianism and internal friction of this profession make me see no hope. I want to find a more humanistic job." | Teaching Adjustment and Optimization |  |
| "When encountering a little problem, I tend to doubt myself, feel that I am not capable enough, and even think about changing my major or career direction." | Task Management and Planning |  |
| "The relationship between internship peers is a naked competitive relationship. Everyone is afraid that others will surpass themselves, so they rarely share experience, and the atmosphere is particularly cold." | Interpersonal Communication Improvement |  |
| "When encountering problems that I can't solve by myself, I take the initiative to consult the supervisor teacher, who will give me specific suggestions and methods." | Proactive Resource Acquisition |  |
| "I will go to the court to play ball to vent my emotions. After sweating a lot, the pressure will be relieved a lot." | Emotional Catharsis and Release | Emotion-Focused Coping |
| "Give myself positive hints, telling myself that it's just lack of experience, and don't deny everything because of one poorly taught class." | Cognitive Restructuring and Regulation |  |
| "Listen to soothing music or dance to music for a while to relax myself." | Leisure and Distraction |  |
| "Write a simple summary to sort out the chaotic ideas. Even a few lines can help me sort out my thoughts quickly." | Writing Reflection and Sorting |  |
| "Call my parents to listen to their encouragement. Family support is a very important spiritual pillar for me." | Social Support Seeking |  |
| "After that class, I was exhausted and felt extremely frustrated. I thought I couldn't even organize a simple small game, so maybe I wasn't qualified to be a physical education teacher at all. I didn't have the energy to teach for several days." | Giving-Up Tendency | Negative Coping |
| "The utilitarianism and internal friction of this profession make me see no hope. I want to find a more humanistic job and no longer want to insist on being a physical education teacher." | Self-Denial |  |
| "When under great pressure, I become particularly emotionally sensitive and cry over small things, but I don't want to tell anyone and just keep it to myself." | Emotional Suppression |  |
| "I was always afraid of saying the wrong thing when communicating with the supervisor teacher, so I tried to speak as little as possible, rarely consulted, and took on problems by myself. Gradually, the relationship with the teacher became very distant." | Interpersonal Alienation |  |
| "I have become more calm and patient, no longer as impetuous as before. My classroom organization and communication skills have also improved a lot." | Teaching Ability Improvement | Positive Adaptation Outcomes |
| "I found that physical education teachers are not only transmitters of sports skills, but also guides for students' growth, who need to balance teaching and educating people." | Professional Cognition Deepening |  |
| "My mentality has become more mature. From avoiding problems to solving them calmly, my self-confidence has increased, and my professional attitude has changed from 'giving it a try' to 'wanting to do it well'." | Mature Mental Model |  |
| "Seeing the students' progress and feeling needed, I have established a solid professional self-confidence and believe that I can become a qualified physical education teacher." | Professional Identity Enhancement |  |
| "Established good relationships with supervisor teachers and internship peers, and can support and help each other when encountering problems." | Harmonious Interpersonal Relationships | Negative Adaptation Outcomes |
| "The utilitarianism and internal friction of this profession make me see no hope. I want to find a more humanistic job." | Professional Burnout Tendency |  |
| "When encountering a little problem, I tend to doubt myself, feel that I am not capable enough, and even think about changing my major or career direction." | Low Self-Efficacy |  |
| "The relationship between internship peers is a naked competitive relationship. Everyone is afraid that others will surpass themselves, so they rarely share experience, and the atmosphere is particularly cold." | Interpersonal Alienation and Avoidance |  |
